# Notch Ligand Delta-Like 1 Is Associated With Loss of Vascular Endothelial Barrier Function

**DOI:** 10.3389/fphys.2021.766713

**Published:** 2021-12-10

**Authors:** Maximilian Moll, Konrad Reichel, Dennis Nurjadi, Sandra Förmer, Lars Johannes Krall, Klaus Heeg, Dagmar Hildebrand

**Affiliations:** Medical Microbiology and Hygiene, Center for Infectious Diseases, Heidelberg University Hospital, Heidelberg, Germany

**Keywords:** infection, sepsis, Notch signaling, DLL1, vascular endothelium, HUVECs, vascular leakage

## Abstract

Vascular leakage associated with vascular endothelial cell (vEC) dysfunction is a hallmark of sepsis. Causative for the decreased integrity of the vascular endothelium (vE) is a complex concurrence of pathogen components, inflammation-associated host factors, and the interaction of vECs and activated circulating immune cells. One signaling pathway that regulates the integrity of the vE is the Notch cascade, which is activated through the binding of a Notch ligand to its respective Notch receptor. Recently, we showed that the soluble form of the Notch ligand Delta-like1 (sDLL1) is highly abundant in the blood of patients with sepsis. However, a direct connection between DLL1-activated Notch signaling and loss of vEC barrier function has not been addressed so far. To study the impact of infection-associated sDLL1, we used human umbilical vein cells (HUVEC) grown in a transwell system and cocultured with blood. Stimulation with sDLL1 induced activation as well as loss of endothelial tight structure and barrier function. Moreover, LPS-stimulated HUVEC activation and increase in endothelial cell permeability could be significantly decreased by blocking DLL1-receptor binding and Notch signaling, confirming the involvement of the cascade in LPS-mediated endothelial dysfunction. In conclusion, our results suggest that during bacterial infection and LPS recognition, DLL1-activated Notch signaling is associated with vascular permeability. This finding might be of clinical relevance in terms of preventing vascular leakage and the severity of sepsis.

## Introduction

The vascular endothelium (vE) constitutes a single layer of cells that line the inner surface of blood vessels as a semipermeable barrier between blood and tissue (Wettschureck et al., 2019). Besides serving as a barrier, the vE has various functions such as maintaining sufficient blood supply of organs through different mechanisms such as prevention of coagulation and regulation of the vascular tone. Additionally, it actively participates in the immune response toward infection (Shao et al., 2020).

Vascular endothelial cells (vECs) do express pathogen recognition receptors, such as Toll-like receptors (TLR), and recognize pathogens during bloodstream infection and components of pathogens that reach the bloodstream from a local infection (Pryshchep et al., 2008; Khakpour et al., 2015). Once activated, vECs produce chemokines that attract immune cells and adhesion molecules enabling the attachment of immune cells to vECs. In parallel, the permeability of the monolayer increases, which allows the transmigration of attached immune cells toward the side of infection (Khakpour et al., 2015).

During sepsis, the function of the vE is highly disturbed (Lee and Slutsky, 2010; Ince et al., 2016). Pathogen-derived components, such as lipopolysaccharide (LPS), and host-derived inflammatory cytokines, such as interleukin (IL)-6 and tumor necrosis factor (TNF)-α, activate endothelial cells and subsequently disrupt the barrier integrity. Furthermore, activated and adhered immune cells, such as neutrophils, directly reinforce permeability (Fox et al., 2013).

One pathway that is involved in the development and maintenance of vE integrity is the Notch signaling cascade (Shawber et al., 2003). This cascade is a conserved signaling pathway and its activation depends on the binding of a transmembrane Notch ligand such as Delta-like1 (DLL1) to a Notch receptor [27]. Ligand binding triggers a series of proteolytic events mediating the cleavage of the extracellular ligand as well as the receptor domain by a disintegrin and metalloproteinase (ADAM) proteases (Radtke et al., 2010). The subsequent cleavage by a γ-secretase release the receptor intracellular domain (NICD), which translocates to the nucleus and mediates gene expression. Notch signaling regulates many cellular developmental processes and the maintenance of organs (Bray, 2016).

The vECs express Notch receptors and activated Notch signaling are crucial for the development of the vasculature (Shawber et al., 2003). Some studies investigated the role of DLL4 and Jagged 1 on the permeability of endothelial cells. They proposed a reduction in permeability (Polacheck et al., 2017; Boardman et al., 2019) as well as loss of integrity through disruption of endothelial adherens junctions (Miloudi et al., 2019). Still, the role of DLL1-induced Notch signaling in vascular leakage during infection is not investigated.

It was shown that several bacterial and some viral infections induce upregulation of DLL1 in the host (Mehrotra et al., 2012; Li et al., 2015; Hildebrand et al., 2018). In patients with sepsis, the elevation of the soluble Notch ligand DLL1 in the blood is particularly pronounced (Hildebrand et al., 2019). Based on these observations, we investigated the impact of DLL1-activated Notch signaling on endothelial cell integrity, especially in the context of LPS activation. We utilized human umbilical vein cells (HUVECs) grown on collagen-coated filters in transwells and cocultured with blood as a well-established vEC model system.

## Materials and Methods

### Healthy Donors

All subjects involved in the study were between the age of 18 and 62, healthy, did not suffer from any illness, and were not pregnant (all signed a questionnaire). Male and female donors were equally distributed across the study.

### Erythrocytes-Depleted-Whole-Blood Retrieval

Blood from healthy donors was taken in a heparin flask. Red blood cell (RBC) lysis-buffer (BD Bioscience, Heidelberg, Germany) was added and incubated for 15 min at room temperature. After centrifugation (1,300 rpm, 10 min), the supernatant was discarded to remove lysed RBCs, and the pellet was resuspended in an endothelial cell growth medium (PromoCell, Heidelberg, Germany).

### Cell Culture

Human umbilical vein cells (PromoCell, Heidelberg, Germany) were seeded (3 × 10^4^ cells per well) in endothelial cell growth medium (PromoCell, Heidelberg, Germany) on the filter of collagen-coated transwells (Corning, Kaiserslautern, Germany) for 8 days (37°C and 5% CO_2_) until a confluent monolayer was formed. Complete formation of the monolayer was confirmed by transendothelial electrical resistance (TEER) measurement. Then, medium from the upper well was removed, and erythrocytes-depleted-whole-blood (edWB) was added to mimic the physiological condition of a blood vessel and naturally occurring contact to blood cells.

### Treatment of Cells

The HUVECs/edWB coculture was stimulated with 5 μg/ml sDLL1 (Pe-Protech, Hamburg, Germany) or 100 ng/ml LPS (Invivogen, Toulouse, France). For inhibition of Notch, signaling cells were pretreated for 1 h with 2.5 μM DAPT (Sigma-Aldrich, Taufkirchen, Germany). For blocking DLL1 binding, 5 μg/ml anti-DLL1 antibody (AbD3593, Bio-Rad, Puchheim, Germany) or 5 μg/ml anti-Notch1 antibody (BioLegend GmbH, Koblenz, Germany) was added 1 h before stimulation.

### *In vitro* Infection

*Escherichia coli* isolate was obtained from a rectal swab of a patient of University Hospital Heidelberg during routine diagnostics. *E. coli* was cultured overnight on Columbia blood sheep agar at 37°C at 5% CO_2_ in a humidified atmosphere. The following day one colony of culture was transferred into Tryptic Soy Broth media and cultured at constant shaking at 200 rpm/37°C until mid-log phase. Then, bacterial suspension was adjusted using absorption measurement. Coculture was infected with multiplicity of infection (MOI) 50. After 2 h of infection, bacteria were killed with gentamicin (Fisher Scientific, Schwerte, Germany).

### Flow Cytometry

**FIGURE 1:**
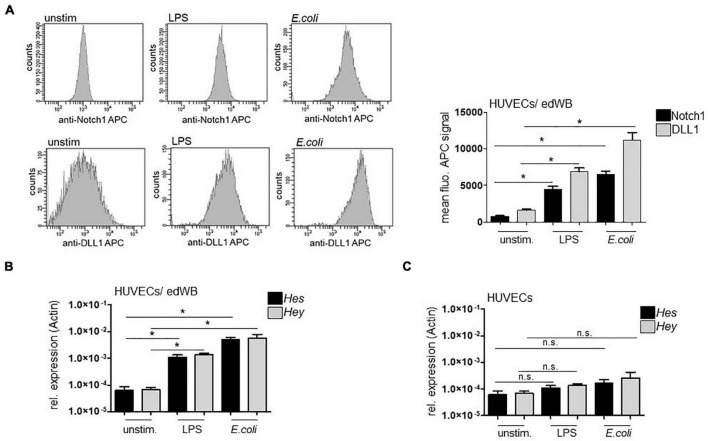
Bacterial stimulation induces Notch1 receptor and DLL1 expression on HUVECs. The 3 × 10^4^ HUVECs were seeded in collagen-coated transwells and grown for 8 days until they formed a monolayer. The 500 μl erythrocyte-depleted whole blood (edWB) was added per well. The HUVECs/edWB coculture was stimulated with 100 ng/ml LPS or an *E. coli* strain (MOI 50) overnight. Then, edWB was removed *via* repeated washing of the endothelial monolayer with PBS. **(A)** HUVECs were harvested through trypsinization, stained with anti-Notch1 receptor antibody and anti-DLL1 antibody, and analyzed by flow cytometry. Left: Histograms of flow cytometry analysis. Right: Quantification of mean fluorescence of bound antibody. **(B,C)** RNA was isolated from HUVECs, previously cocultured with edWB **(B)** or without edWB **(C)** and cDNA was produced. Quantitative rt-PCR was performed with SYBR Green Rox Mix and sequence specific primers for *Hes*1 and *Hey*1. Gene expression was normalized against the actin housekeeping gene (relative expression). Data were analyzed using Prism9 software (mean ± standard deviation; *n* = 3) and significance was calculated using a one-sided Mann-Whitney *U*-test. *p ≤ 0.05, n.s. = not significant.

**FIGURE 2:**
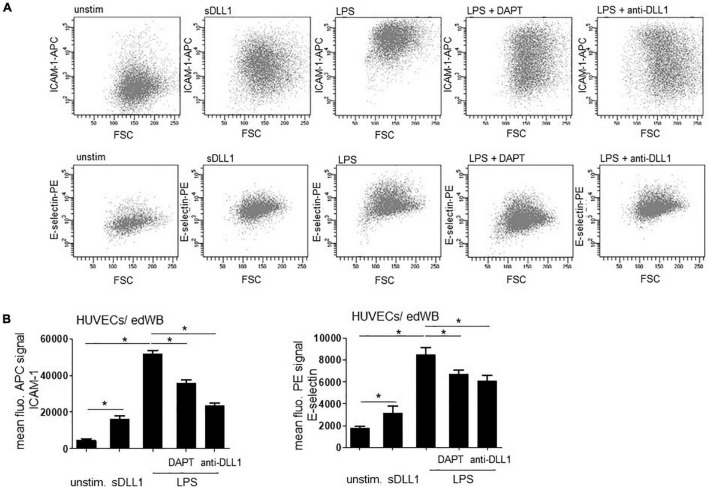
Notch signaling promotes LPS-mediated endothelial cell activation. HUVECs/edWB coculture was stimulated with sDLL1 (5 μg/ml), LPS (100 ng/ml), LPS + DAPT (1 μM, pretreatment for 1 h), or LPS + anti-DLL1 antibody (5 μg/ml) overnight. After removing blood and repeated washing, HUVECs were harvested and stained with anti-ICAM-1 antibody and anti-E-selectin antibody and analyzed by flow cytometry. **(A)** Dot blots of flow cytometry analysis. **(B)** Quantification of mean fluorescence of bound antibody (mean ± standard deviation; *n* = 3). Statistics: Prism9 software, one-sided Mann-Whitney *U*-test. *p ≤ 0.05.

The HUVECs/edWB coculture was stimulated with 100 ng/ml LPS or an *E. coli* strain (MOI 50) overnight (Figure 1) or with sDLL1 (5 μg/ml), LPS (100 ng/ml) ± DAPT (1 μM, pretreatment for 1 h), or LPS + anti-DLL1 antibody (5 μg/ml) overnight (Figure 2). After removing edWB and rinsing with phosphate-buffered saline (PBS), HUVECs were analyzed for surface expression of DLL1 (anti-DLL1-APC, Miltenyi Biotec, Bergisch Gladbach, Germany), Notch1 receptor (anti-Notch1-APC, BioLegend GmbH, Koblenz, Germany), ICAM1 (anti-CD54-APC), and E-selectin (anti-CD62E-PE) (both BD Bioscience, Heidelberg, Germany) with antibody staining and flow cytometry. Fluorescence was recorded using the FACS DIVA V 4.12 software on a FACS Canto (BD Biosciences, Heidelberg, Germany).

### Quantitative Reverse Transcription PCR

The HUVECs with or without edWB were stimulated with 100 ng/ml LPS or an *E. coli* strain (MOI 50) overnight. Blood cells were removed from the coculture by repeated washing with PBS. Total RNA was extracted from HUVECs using the High Pure RNA Isolation Kit (Roche, Mannheim, Germany) according to the protocol of the manufacturer, and cDNA was produced using ReverseAid First Strand cDNA Synthesis Kit (Thermo Fischer Scientific, Karlsruhe, Germany). The cDNA obtained was used for quantitative PCR utilizing the “SYBR Green ROX Mix” (Thermo Fischer Scientific, Karlsruhe, Germany) and the following sequence-specific primers: *actin* forward 5′-AGA GCT ACG AGC TGC CTG AC-3′, *actin* reverse 5′-AGC ACT GTG TTG GCG TAC AG-3′, *HES1* forward 5′-CTGAAGAAAGATAGCTCGCG-3′, *HES1* reverse 5′-ACTTCCCCAGCACACTT-3′, *HEY1* forward 5′-AGCCGAGATCCTGCAAGATGA-3′, and *HEY1* reverse 5′-GCCGTATGCAGCATTTTCAG-3′.

### Transendothelial Electrical Resistance

The HUVECs with or without edWB were stimulated with 100 ng/ml LPS or 5 μg sDLL1 or LPS + anti-DLL1 antibody (5 μg/ml), LPS + anti-Notch1 antibody (5 μg/ml), and LPS + DAPT (1 μM pretreatment for 1 h) for 24 h. The TEER was measured using a volt-ohm meter EVOM^2^ and an STX1 electrode (WPI, Friedberg, Germany) following the protocol of the manufacturers after 0, 0.5, 1, 3, and 24 h. Data were calculated by subtracting resistance values of a transwell without cells and multiplied with the surface area (1.12 cm^2^) of the transwell. To confirm that cell numbers are equal in each condition during the experiment, we harvested cells of all conditions by trypsinization after 24 h and counted trypan blue stained cells.

### Permeability Assay

The HUVECs, cultivated with edWB, were stimulated using 100 ng/ml LPS or 5 μg/ml sDLL1 or LPS + anti-DLL1 antibody (5 μg/ml), LPS + anti-Notch1 antibody (5 μg/ml), and LPS + DAPT (1 μM pretreatment for 1 h). Post stimulation, 15 μg/ml (0.1 nmol/ml) fluorescein isothiocyanate (FITC)-dextran (Sigma Aldrich, Taufkirchen, Germany) was added to the upper chamber of each well. After 0, 0.5, 1, 3, and 24 h, 50 μl from the lower chamber of each well was transferred into a Black 96 Well Microplate (Greiner Bio-One, Frickenhausen, Germany) and Black 96-Well Plate Reader (LUMIstar OPTIMA, BMG LABTECH, Ortenberg, Germany) and measured at 488 nm excitation and 555 nm emission. In addition, a serial dilution of FITC-dextran was added to the plate in each experiment as a standard for the calculation of dextran concentration.

### Immunofluorescence Imaging

Coculture was stimulated with sDLL1 (5 μg/ml) for 3 h or left unstimulated. After removing edWB, the HUVECs were fixed (2% PFA in PBS, 30 min at RT) on the transwell membrane and permeabilized with 0.1% Triton-X in PBS (TX-PBS) for 15 min at RT. Non-specific binding sites were blocked with TX-PBS 5.5% FCS for 1 h at RT. Staining was performed with primary antibody (45 min) against VE-Cadherin and secondary antibody (1 h) anti-mouse-Cy5 or anti-ZO-1 (all three Thermo Fischer Scientific, Karlsruhe, Germany) and secondary antibody anti-rabbit-DyLight488 (Abcam, Cambridge, United Kingdom). All antibodies were diluted in PBS to a final concentration of 5 μg/ml. Actin was visualized later with phalloidin-tetramethylrhodamine B isothiocyanat, 1:2,000 in PBS for 45 min (Sigma Aldrich, Taufkirchen, Germany). DNA was stained (20 min) with Hoechst-33342, 1:10,000 in PBS (Thermo Fischer Scientific, Karlsruhe, Germany). Membranes with stained cells were transferred to glass slides and mounted on a microscope (ROTI^®^Mount Aqua, Carl Roth GmbH + Co., KG, Karlsruhe, Germany). Images were taken using a Leica SP8 confocal microscope and LAS X software (Leica Microsystems GmbH, Wetzlar, Germany). Pictures were analyzed, processed, and quantified in Fiji/ImageJ (Wayne Rasband). For quantification of Ve-Cadherin and ZO-1, microscopic images were converted into black and white, and integrated density was measured from total cells and from the cytoplasm of cells. Then, corrected total fluorescence (CTCF) was calculated using the following formula: CTCF = Integrated density – (Area of selected cell × Mean fluorescence of background readings).

### Statistical Analysis

Statistical analysis was performed using Graphpad Prism version 9 (San Diego, California, United States). Values were presented as mean with standard deviation. Comparison of quantitative variables between groups was calculated using the non-parametric Mann-Whitney *U*-test. The level of significance was set at a *p*-value ≤ 0.05.

### Ethics Statement

This study was carried out in accordance with the recommendations of the ethics committee of the Medizinische Fakultät, Heidelberg (S-157/2006). All subjects gave written informed consent in accordance with the Declaration of Helsinki.

## Results

### Stimulation With *Escherichia coli* and Lipopolysachharide Induces Notch Signaling in Endothelial Cells

First, we assessed the ability of HUVECs to respond to bacterial stimulation with upregulation of DLL1 and DLL1-receptor Notch1. The HUVECs/edWB coculture was stimulated with LPS or infected with an *E. coli* strain (MOI 50) and analyzed using antibody staining and flow cytometry for the induction of DLL1 and Notch1 receptor surface expression. LPS stimulation and *in vitro* infection with *E. coli* triggered the expression of ligand and receptor significantly (Figure 1A). Then, we confirmed that bacterial stimulation induced Notch signaling by performing quantitative real-time (rt)-PCRs for Notch target genes *Hes1* and *Hey1*. LPS as well as *E. coli* stimulation induced a pronounced gene induction of both target genes (Figure 1B). Stimulation of HUVECs without edWB induced only a minor induction of target genes implicating the need for activated blood cells (Figure 1C). Having shown that whole *E. coli* activates Notch signaling in HUVECs, the following experiments were performed exclusively with LPS as one major stimulatory component of gram-negative bacteria such as *E. coli*.

### Notch Signaling Promotes Lipopolysaccharide-Mediated Endothelial Cell Activation

To verify the involvement of Notch signaling in LPS-stimulated vEC activation, HUVECs/edWB coculture was stimulated with LPS and pretreated with Notch inhibitor DAPT. Flow cytometry analysis revealed that LPS stimulation significantly induced the endothelial activation marker ICAM-1 and E-selectin, as expected. Inhibition of the Notch cascade through DAPT diminished the expression significantly, implicating the involvement of Notch signaling in LPS-induced endothelial cell activation. Importantly, pretreatment with an anti-DLL1 blocking antibody also dampened the LPS-induced activation, implicating a role for DLL1-induced Notch signaling (Figures 2A,B). To further investigate the impact of DLL1, the coculture was stimulated with recombinant soluble DLL1 (sDLL1), which significantly upregulated ICAM-1 as well as E-selectin on HUVECs (Figures 2A,B).

### DLL1-Induced Notch Signaling Dampens Integrity of HUVEC Monolayer

**FIGURE 3:**
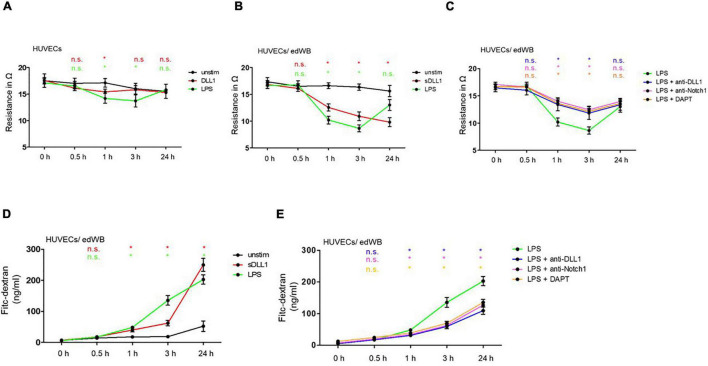
Notch signaling participates in LPS-mediated vE permeability. HUVECs cultivated alone **(A)** or in coculture with edWB **(B,C)** were stimulated with 100 ng/ml LPS or 5 μg/ml sDLL1 **(B)**, or LPS + anti-DLL1 antibody (5 μg/ml), anti-Notch antibody (5 μg/ml), DAPT (1 μM pretreatment for 1 h) for 24 h **(C)**. TEER was measured with the EVOM2 volt/ohm-meter after 0, 0.5, 1, 3, and 24 h. **(D,E)** HUVECs were cocultured with edWB and stimulated with 100 ng/ml LPS or 5 μg/ml sDLL1 **(D)** or LPS + anti-DLL1 antibody (5 μg/ml), LPS + anti-Notch1 antibody (5 μg/ml), DAPT (1 μM pretreatment for 1 h). **(E)** FITC-dextran was added on top of the transwells, and fluorescence was measured *via* a fluorescence reader after 0, 0.5, 1, 3, and 24 h in aliquots taken from the lower chamber. Data were analyzed using Prism9 and significance was calculated using a one-sided Mann-Whitney *U*-test. *p ≤ 0.05. Red, significance of sDLL1 vs. unstimulated; green, significance of LPS vs. unstimulated; blue, significance of LPS + anti-DLL1 vs. LPS; pink, significance of LPS + anti-Notch1 vs. LPS; orange, significance of LPS + DAPT vs. LPS.

To evaluate the impact of Notch signaling on the integrity of vascular endothelial monolayer, the barrier function of HUVECs was analyzed. First, the integrity of HUVECs was determined by TEER measurement across the cellular monolayer. Post stimulation, TEER was measured at different time points. LPS stimulation of HUVECs without edWB induced a minor, although significant, decrease in resistance after 1 and 3 h [Δ mean 1 h (3 h) in Ω: 2.93 (2.30); both *p* = 0.05*]. Similar finding was observed for stimulation with sDLL1 [Δ mean 1 h (3 h) in Ω: 0.9 (1.7); *p* = 0.05* (*p* = 0.412)] (Figure 3A). However, in the HUVECs/edWB coculture, LPS stimulation induced a pronounced decrease in resistance of HUVEC monolayer after 1 h (Δ mean in Ω: 6.4: *p* = 0.05*) that stayed stable up to 3 h (Δ mean in Ω: 7.1; *p* = 0.05*), confirming once again that blood cells are important in terms of loosening endothelial cell structure. After 24 h, the decrease in resistance faded (Δ mean in Ω: 2.6; *p* = 0.06), and the integrity began to stabilize again. sDLL1 mediated a distinct decrease in TEER after 1 h of stimulation (Δ mean in Ω: 4.07; *p* = 0.05*). This effect was stable for 24 h and slightly increased further during that time (Δ mean in Ω: 6.80; *p* = 0.05*) (Figure 3B). Importantly, blocking DLL1/receptor binding *via* an anti-DLL1 antibody and an anti-Notch1 antibody diminished the LPS-induced decrease in resistance significantly after 1 and 3 h [anti-DLL1: Δ mean 1 h (3 h) in Ω: 3.2 (2.93); anti-Notch1: Δ mean 1 h (3 h) in Ω: 3.1 (3.6); all *p* = 0.05*]. The same was true for inhibiting Notch signaling *via* DAPT (Δ mean 1 h [3 h] in Ω: 2.9 (4.2); both *p* = 0.05*] (Figure 3C).

Next, fluorescence-based transwell assays were performed. FITC-dextran was added on top of the transwells. Over time, transmigration of dextran in the lower well was analyzed. Comparable to the TEER measurement, transmigration of dextran started after 1 h of LPS and DLL1 stimulation. sDLL1 increased transmigration around 3-fold after 3 h of stimulation and almost 5-fold after 24 h. LPS-mediated transmigration of dextran increased significantly more than 6-fold after 3 h and stabilized to around 4-fold after 24 h (Figure 3D). Importantly, anti-DLL1 antibody, anti-Notch1 antibody, and DAPT could dampen the LPS-associated transmigration of dextran almost by half (Figure 3E). According to these results, DLL1-mediated Notch signaling mediates an increase in the permeability of HUVEC monolayers.

**FIGURE 4:**
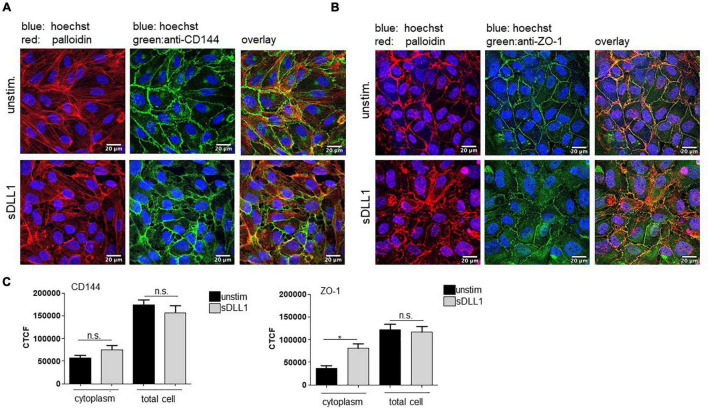
sDLL1 promotes loosening of HUVEC structure. Coculture was stimulated with sDLL1 (5 μg/ml) for 3 h. Blood cells were removed *via* rinsing with PBS. HUVECs were fixed on membranes with PBS/PFA and permeabilized with PBS/Triton-X. Cells were stained with anti-CD144 against VE-cadherin and anti-mouse-cy5 [**(A)**, shown in green], or anti-Zo-1 and anti-rabbit-DyLight488 [**(B)**, shown in green], phalloidin-tetramethylrhodamine B Isothiocyanat against F-actin (shown in red), and Hoechst-33342 against DNA (shown in blue). Membranes were cut and mounted on microscopic slides. Images were taken using a Leica SP8 confocal microscope, an inverted microscope, and LAS X software. Images were analyzed and processed in Fiji/ImageJ. **(C)** For quantification of Ve-cadherin and ZO-1, microscopic images were converted into black and white, and integrated density was measured from total cells and the cytoplasm of cells. Quantifications depict corrected total fluorescence (CTCF). n.s. = not significant. The experiment was repeated three times with comparable results.

We further characterized the effect of sDLL1 and Notch signaling on endothelial cell integrity by antibody staining and confocal microscopy imaging techniques. Cells were analyzed for morphological changes and expression and localization of VE-cadherin, the major component of adherens junctions. Furthermore, ZO-1, as an important regulator of adherens junctions and barrier stability, was analyzed. Stimulation with sDLL1 for 3 h clearly modulated the structure of the HUVEC monolayer. The tight structure that can be observed in the image of the unstimulated sample started to loosen and gaps began to form between cells (Figure 4). In the unstimulated HUVECs, VE-cadherin (CD144) (Figure 4A) and ZO-1 (Figure 4B) locate mostly in the plasma membrane, showing the tight cohesion between single cells. However, stimulation with sDLL1 seems to mediate the translocation of the proteins into the cytoplasm. The increased appearance in the cytoplasm is significant for ZO-1 and not significant but replicable for VE-cadherin (Figures 4A–C).

## Discussion

In this study, we presented evidence that DLL1-activated Notch signaling promotes loss of vECs integrity and participates in LPS-mediated vEC activation and subsequent loss of vEC barrier function.

Endothelial dysfunction and vascular leakage are hallmarks of sepsis, and activation markers such as E-selectin are considered as predictive markers for sepsis severity (Shapiro et al., 2010; Ince et al., 2016). During infection, the vE actively participates in the immune response (Shao et al., 2020). The vECs are activated by bacterial components, such as LPS, and host factors, such as cytokines and chemokines, during infection. Activation leads to upregulation and exposition of adhesin molecules and increased leukocyte adhesion, enhanced permeability, and transmission of immune cells into surrounding tissue (Shapiro et al., 2010; Shao et al., 2020). In combination with increased coagulation and an altered vasomotor tone, these modulations allow combating local infection effectively. In sepsis, conditioned by a hyperactivated immune system, the process of vascular alteration might become out of balance leading to microcirculatory disturbances, impaired perfusion, tissue hypoxia, and organ failure (Ince et al., 2016).

The Notch signaling pathway highly impacts development as well as stability of the vascular (Shawber et al., 2003; Scheppke et al., 2012), and the Notch ligand DLL1 is highly upregulated in patients with sepsis (Hildebrand et al., 2019). Nevertheless, the role of the Notch cascade in infection and sepsis-associated vE dysfunction remains unknown. The majority of studies on Notch-regulated vascular barrier function are associated with hyperglycemic conditions in diabetes models. In previous studies, upregulated Jagged1 and DLL4 are accused to facilitate conditions associated with diabetes-induced vascular permeability such as diabetic retinopathy and diabetic macular edema (Yoon et al., 2016; Miloudi et al., 2019). In another simplified *in vitro* approach with soluble DLL4-activated human dermal blood endothelial cells, DLL4 stabilizes the integrity of the endothelial cells (Boardman et al., 2019). The role of DLL1 is not addressed in either of these studies, and the effects of one Notch ligand cannot be transferred to another one. As DLL1 is the Notch ligand that is highly upregulated in patients with sepsis, we concentrated on this ligand.

In this study, we showed that LPS-stimulated and *E. coli*-infected HUVECs cocultured with edWB do upregulate transmembrane DLL1 and Notch1 receptor and, subsequently, induce Notch target gene induction. According to our data, blood cells are mandatory for a pronounced activation of the Notch pathway. Whether blood cells do mediate the upregulation of TLRs and/or Notch receptors cannot be answered with our study. However, this hypothesis seems likely. Activation of Notch correlated with an increase in HUVEC activation, loss of endothelial tight structure, and loss of barrier function. Again, the decrease in vEC integrity is largely dependent on coculture with whole blood. Importantly, the LPS-mediated effects could be significantly decreased by blocking DLL1-receptor binding and Notch signaling, confirming the involvement of the cascade. Most likely, blood cells are needed to induce a sufficient production of DLL1 in HUVECs and blood cells for a high Notch cascade activation. The evidence for the importance of DLL1-induced Notch signaling could be further strengthened by stimulation of cells with sDLL1 exclusively. In fact, DLL1 alone was able to induce activation as well as a profound increase in permeability of the HUVEC monolayer. Stimulation induced distinct gaps between vECs. This loss of tight endothelial structure was associated with displacement of ZO-1 and VE-cadherin from the cell membrane to the interior of the cell. VE-cadherin is the major component of adherens junctions. It tightly regulates protein complexes that interlink adjacent endothelial cells and prevent leukocyte emigration and vascular leakage. The translocation of VE-cadherin from the cell membrane to the cytoplasm of the cell is sufficient to induce gaps between endothelial cells, causing increased permeability (Gotsch et al., 1997; Corada et al., 1999; Vestweber, 2008).

Our finding might not only be relevant in terms of bacterial infection and sepsis. In severe dengue fever (DF) manifestations, plasma leakage and bleeding is considered one of the key features (Khetarpal and Khanna, 2016). In fact, dengue virus infection has been shown to induce upregulation of DLL1 although to a less extent than observed in sepsis (Li et al., 2015). Interestingly, in DF, we could show that sDLL1 levels in the blood of patients are associated with bleeding manifestation (Hildebrand et al., 2021). Together with the data presented here, one could speculate that DLL1 might be involved in the pathology of DF.

Our study utilizes an *in vitro* model system. Future studies could evaluate our findings in a mouse model of LPS-induced systemic inflammation.

## Conclusion

We provided evidence that DLL1-activated Notch signaling, as occurring in severe infections, is associated with the loss of vEC barrier function. Therefore, Notch signaling and DLL1 might be an interesting therapeutic target to prevent vascular leakage and severe progression in infectious diseases such as sepsis.

## Data Availability Statement

The original contributions presented in the study are included in the article/supplementary material, further inquiries can be directed to the corresponding author/s.

## Ethics Statement

The studies involving human participants were reviewed and approved by Medical Faculty Heidelberg, University Heidelberg. The patients/participants provided their written informed consent to participate in this study.

## Author Contributions

DH designed the study with essential contributions from KH, MM, KR, and DN. MM and KR performed a majority of the experiments and analyses. SF conducted experiments. DN provided the *E. coli* strain and supported the analyses. LJK supported with TEER expertise. DH, MM, and DN prepared the manuscript. All authors discussed the results and implications and approved the manuscript.

## Conflict of Interest

The authors declare that the research was conducted in the absence of any commercial or financial relationships that could be construed as a potential conflict of interest.

## Publisher’s Note

All claims expressed in this article are solely those of the authors and do not necessarily represent those of their affiliated organizations, or those of the publisher, the editors and the reviewers. Any product that may be evaluated in this article, or claim that may be made by its manufacturer, is not guaranteed or endorsed by the publisher.

## References

[B1] Boardman R. Pang V. Malhi N. Lynch A. P. Leach L. Benest A. V. (2019). Activation of Notch signaling by soluble Dll4 decreases vascular permeability via a cAMP/PKA-dependent pathway. *Am. J. Physiol. Heart Circ. Physiol.* 316 H1065–H1075. 10.1152/ajpheart.00610.2018 30681366 PMC6580391

[B2] Bray S. J. (2016). Notch signalling in context. *Nat. Rev. Mol. Cell Biol.* 17 722–735. 27507209 10.1038/nrm.2016.94

[B3] Corada M. Mariotti M. Thurston G. Smith K. Kunkel R. Brockhaus M. (1999). Vascular endothelial-cadherin is an important determinant of microvascular integrity in vivo. *Proc. Natl. Acad. Sci. U.S.A.* 96 9815–9820. 10449777 10.1073/pnas.96.17.9815 PMC22293

[B4] Fox E. D. Heffernan D. S. Cioffi W. G. Reichner J. S. (2013). Neutrophils from critically ill septic patients mediate profound loss of endothelial barrier integrity. *Crit. Care* 17:R226. 10.1186/cc13049 24099563 PMC4057230

[B5] Gotsch U. Borges E. Bosse R. Boggemeyer E. Simon M. Mossmann H. (1997). VE-cadherin antibody accelerates neutrophil recruitment in vivo. *J. Cell Sci.* 110(Pt 5), 583–588. 10.1242/jcs.110.5.583 9092940

[B6] Hildebrand D. Decker S. O. Koch C. Schmitt F. C. F. Ruhrmann S. Schneck E. (2019). Host-derived delta-like canonical notch ligand 1 as a novel diagnostic biomarker for bacterial sepsis-results from a combinational secondary analysis. *Front. Cell Infect. Microbiol.* 9:267. 10.3389/fcimb.2019.00267 31396491 PMC6663974

[B7] Hildebrand D. Uhle F. Sahin D. Krauser U. Weigand M. A. Heeg K. (2018). The interplay of notch signaling and STAT3 in TLR-activated human primary monocytes. *Front. Cell Infect. Microbiol.* 8:241. 10.3389/fcimb.2018.00241 30042932 PMC6048282

[B8] Hildebrand D. Nurjadi D. Hoan N. X. Linh M. T. H. Sang V. V. Bang M. H. (2021). Soluble notch ligand DLL1 is associated with bleeding complication in patients with dengue fever infection. *J. Infect. Dis.* jiab404. 10.1093/infdis/jiab404 [Epub ahead of print]. 34375432

[B9] Ince C. Mayeux P. R. Nguyen T. Gomez H. Kellum J. A. Ospina-Tascon G. A. (2016). The endothelium in sepsis. *Shock* 45 259–270. 26871664 10.1097/SHK.0000000000000473 PMC5281063

[B10] Khakpour S. Wilhelmsen K. Hellman J. (2015). Vascular endothelial cell Toll-like receptor pathways in sepsis. *Innate Immun.* 21 827–846. 10.1177/1753425915606525 26403174

[B11] Khetarpal N. Khanna I. (2016). Dengue fever: causes, complications, and vaccine strategies. *J. Immunol. Res.* 2016:6803098. 27525287 10.1155/2016/6803098 PMC4971387

[B12] Lee W. L. Slutsky A. S. (2010). Sepsis and endothelial permeability. *N. Engl. J. Med.* 363 689–691. 10.1056/nejmcibr1007320 20818861

[B13] Li Y. Wu S. Pu J. Huang X. Zhang P. (2015). Dengue virus up-regulates expression of notch ligands Dll1 and Dll4 through interferon-beta signalling pathway. *Immunology* 144 127–138. 10.1111/imm.12357 25041739 PMC4264916

[B14] Mehrotra S. Fakiola M. Mishra A. Sudarshan M. Tiwary P. Rani D. S. (2012). Genetic and functional evaluation of the role of DLL1 in susceptibility to visceral leishmaniasis in India. *Infect. Genet. Evol.* 12 1195–1201. 10.1016/j.meegid.2012.04.017 22561395 PMC3651914

[B15] Miloudi K. Oubaha M. Ménard C. Dejda A. Guber V. Cagnone G. (2019). NOTCH1 signaling induces pathological vascular permeability in diabetic retinopathy. *Proc. Natl. Acad. Sci. U.S.A.* 116 4538–4547. 10.1073/pnas.1814711116 30787185 PMC6410871

[B16] Polacheck W. J. Kutys M. L. Yang J. Eyckmans J. Wu Y. Vasavada H. (2017). A non-canonical Notch complex regulates adherens junctions and vascular barrier function. *Nature* 552 258–262. 10.1038/nature24998 29160307 PMC5730479

[B17] Pryshchep O. Ma-Krupa W. Younge B. R. Goronzy J. J. Weyand C. M. (2008). Vessel-specific toll-like receptor profiles in human medium and large arteries. *Circulation* 118 1276–1284. 10.1161/CIRCULATIONAHA.108.789172 18765390 PMC2748975

[B18] Radtke F. Fasnacht N. Macdonald H. R. (2010). Notch signaling in the immune system. *Immunity* 32 14–27. 10.1016/j.immuni.2010.01.004 20152168

[B19] Scheppke L. Murphy E. A. Zarpellon A. Hofmann J. J. Merkulova A. Shields D. J. (2012). Notch promotes vascular maturation by inducing integrin-mediated smooth muscle cell adhesion to the endothelial basement membrane. *Blood* 119 2149–2158. 10.1182/blood-2011-04-348706 22134168 PMC3311249

[B20] Shao Y. Saredy J. Yang W. Y. Sun Y. Lu Y. Saaoud F. (2020). Vascular endothelial cells and innate immunity. *Arterioscler Thromb. Vasc. Biol.* 40 e138–e152. 32459541 10.1161/ATVBAHA.120.314330 PMC7263359

[B21] Shapiro N. I. Schuetz P. Yano K. Sorasaki M. Parikh S. M. Jones A. E. (2010). The association of endothelial cell signaling, severity of illness, and organ dysfunction in sepsis. *Crit Care* 14:R182. 10.1186/cc9290 20942957 PMC3219288

[B22] Shawber C. J. Das I. Francisco E. Kitajewski J. (2003). Notch signaling in primary endothelial cells. *Ann. N, Y, Acad. Sci.* 995 162–170. 12814948 10.1111/j.1749-6632.2003.tb03219.x

[B23] Vestweber D. (2008). VE-cadherin: the major endothelial adhesion molecule controlling cellular junctions and blood vessel formation. *Arterioscler Thromb, Vasc, Biol*, 28 223–232. 10.1161/atvbaha.107.158014 18162609

[B24] Wettschureck N. Strilic B. Offermanns S. (2019). Passing the vascular barrier: endothelial signaling processes controlling extravasation. *Physiol, Rev*, 99 1467–1525. 10.1152/physrev.00037.2018 31140373

[B25] Yoon C. H. Choi Y. E. Cha Y. R. Koh S. J. Choi J. I. Kim T. W. (2016). Diabetes-induced jagged1 overexpression in endothelial cells causes retinal capillary regression in a murine model of diabetes mellitus: insights into diabetic retinopathy. *Circulation* 134 233–247. 10.1161/CIRCULATIONAHA.116.014411 27407072

